# Endocrine profile of the VCD-induced perimenopausal model rat

**DOI:** 10.1371/journal.pone.0226874

**Published:** 2019-12-30

**Authors:** Ruither O. G. Carolino, Paulo T. Barros, Bruna Kalil, Janete Anselmo-Franci

**Affiliations:** 1 Department of Basic and Oral Biology, School of Dentistry of Ribeirão Preto, University of São Paulo, Ribeirão Preto, SP, Brazil; 2 Department of Physiology, School of Medicine of Ribeirão Preto, University of São Paulo, Ribeirão Preto, SP, Brazil; 3 Department of Physiology, Institute of Biomedical Science, Federal University of Alfenas, Alfenas, MG, Brazil; University of Sydney, AUSTRALIA

## Abstract

During the transition to menopause, women experience a variety of physical and psychological symptoms that are directly or indirectly linked to changes in hormone secretion. Establishing animal models with intact ovaries is essential for understanding these interactions and finding new therapeutic targets. In this study, we assessed the endocrine profile, as well as the estrous cycle, in the 4-vinylcyclohexene diepoxide (VCD)-induced follicular depletion rat model in 10-day intervals over 1 month to accurately establish the best period for studies of the transition period. Twenty-eight-day-old female rats were injected daily with VCD or oil s.c. for 15 days and euthanized in the diestrus phase approximately 70, 80, 90 and 100 days after the onset of treatment. The percentage of rats showing irregular cycles and the plasma level of FSH increased only in the 100-day VCD group. Plasma anti-Müllerian hormone (AMH) and progesterone were lower in all VCD groups compared to control groups, while estradiol remained unchanged or higher. As in control groups, dihydrotestosterone (DHT) progressively decreased in the 70-90-day VCD groups; however, it was followed by a sharp increase only in the 100-day VCD group. No changes were found in plasma corticosterone, prolactin, thyroid hormones or luteinizing hormone. Based on the estrous cycle and endocrine profile, we conclude that 1) the time window from 70 to 100 days is suitable to study a perimenopause-like state in this model, and 2) regular cycles with low progesterone and AMH and normal FSH can be used as markers of the early/mid-transition period, whereas irregular cycles associated with higher FSH and DHT can be used as markers of the late transition period to estropause.

## Introduction

Perimenopause is the transition period from the reproductive to the nonreproductive stage of life and includes the last reproductive years through the first twelve consecutive months without a menstrual period [1]. This period corresponds to the final depletion of oocytes and can last up to 10 years, at time women may experience important changes in their hormone secretion [2]. These hormonal changes are usually accompanied by symptoms such as hot flushes, night sweats, sleep alterations, migraines, and mood disorders, such as anxiety and depression [3, 4]. Therefore, it is crucial to identify endocrine alterations that might lead to these physical and psychological disturbances.

Evaluating hormonal changes in perimenopausal women as well as the mechanisms underlying these changes is challenging. While the ideal animal model, the nonhuman primate model, has become increasingly less used because of ethical issues, the naturally aging rat model has its unique characteristics that genuinely differ from the reproductive aging process in humans. Although middle-aged rats, similar to women, progress from regular to irregular/elongated cycles, a period referred to as estropause, they continue to cycle for a long time during senescence, even until death [5, 6]. In addition, according to the current hypothesis, reproductive aging in humans is characterized by a progressive loss in ovarian function, which leads to alterations in sex hormone secretion [7]. On the other hand, reproductive aging in rats seems to begin as a neuroendocrine process that starts independently of the ovarian competence, since irregular cycles and decreased fertility occur without a decrease in the follicular stores [8, 9] and, under appropriate gonadotropic stimulation, the ovaries of old rats 20–30 months old are capable of normal function [10, 11]. Therefore, it seems that the major cause of ovarian loss of function in old rats lies in altered hypothalamus-pituitary function. Due to these differences, ovariectomized rats have been widely used as an animal model for menopause studies. However, the transition period cannot be achieved in this model since the levels of ovarian hormones decrease abruptly after removal of the ovaries, thus eliminating the transitional period.

In this context, there is a pressing need for a reliable animal model that resembles the endocrine milieu of female perimenopause to allow further investigations of the peripheral and central alterations occurring during this transition period of reproductive life. The chemical 4-vinylcyclohexene diepoxide (VCD) was described to induce an acceleration in the depletion of primordial and primary follicles in female rodents [12, 13], so that treating rats in early life with this compound can induce a decrease in ovarian function before the central alterations to occur, thus eliminating one of the main differences between rodents and women. Because in women the primary cause of reproductive aging is the follicular reserve depletion, the VCD animal model resembles the human condition by shifting the primary cause of reproductive senescence from brain to ovary. Thus, it is likely that the ovarian as well hormonal changes in this model are similar to the changes experienced by women during the natural menopause transition. This led us to think that VCD-induced follicular depletion was an attractive animal model for studying the menopause transition.

There are several reports regarding temporal hormone alteration in the VCD animal model, most of them from Dr. Hoyer’s group. However, the majority of them were done in mice [14–19], in which the mechanisms of action of VCD in the ovary differ from those in rats [20]. As shown by Hoyer’s group in mice, follicle destruction occurs earlier and to a greater extent than in rats [21], in consonance with the faster effects of VCD in mice, FSH increases 35 days after the onset of VCD treatment in mice [14], while in rats, it takes 120 days [22].

In rats, studies applying the most commonly used protocol (onset of VCD injections approximately 30 days of age) are scarce and have evaluated the levels of one or more hormones at a single time point [23–25] or over a long time interval of months [22, 26, 27]. We have published recently a study showing that 80 days after the onset of VCD treatment, plasma levels of progesterone and androgens decreased, while no changes were observed in plasma follicle-stimulating hormone (FSH) or estradiol [24]. This hormonal profile is similar to that of early/middle perimenopause [28–30]. However, as our previous study covered only 80 days after the onset of VCD treatment, it did not allow us to accurately evaluate the temporal progression of cycles and hormonal levels in the transition period.

Long-term studies are also not suitable to precisely assess the progression of the transition period induced by VCD. In rats, studies measuring temporal changes in FSH after the onset of VCD treatment found higher levels at 120 days (4.9 mo.) compared to the normal levels at 60 days (1.9 mo.) [22], higher levels at 5.2 mo. compared to 2.0 mo. [26], and higher levels at 5.0 mo. versus 2.4 mo. [27]. Therefore, since FSH was still low at 2.7 mo. [24] and high at 4.9 mo. [22], it may increase sometime between 2.7 and 4.9 mo. Assuming that in women FSH starts to increase in late perimenopause, as described by Burger and colleagues [28], we could predict that in the VCD model late period of the transition to estropause in this animal model might be in this window of time.

In the present study, rats were evaluated every ten days from 70 to 100 days after the onset of VCD treatment, when the rats were 3.2 to 4.2 mo. old, in an attempt to identify when cycles become irregular and FSH levels begin to increase, thus characterizing the turning point of VCD-induced estropause. The hormonal profiles of ten other hormones were also evaluated.

To the best of our knowledge, there is no other report in the literature describing the pattern of several hormones’ secretion exclusively throughout a short and critical period of VCD-induced follicular depletion in rats.

## Materials and methods

### Animals

Twenty-one-day-old female Wistar rats were housed in groups of 4 and maintained in a 12 h/12 h dark/light cycle (lights on at 6:00 a.m.) at 24±0.5°C with standard chow and water provided *ad libitum*. All procedures were approved by the Committee for Animal Care and Use, University of São Paulo, School of Dentistry of Ribeirão Preto (n°. 2013.1.474.58.9).

### VCD administration

The chemical 4-vinylcyclohexene diepoxide (Sigma-Aldrich, Saint Louis, MO) was dissolved in corn oil at a concentration of 128 mg/mL. During VCD treatment, each animal was weighed on alternating days to set the dose of VCD, which was administered daily at a dose of 160 mg/kg subcutaneously over 15 days [24, 25]. Control animals were daily injected with corn oil (1.25 mL/kg, *b*.*w*.*)*. The reason for choosing the subcutaneous route was that it induces minimal deleterious side effects, which can be severe when the intramuscular or intraperitoneal route is used [31].

### Experimental design

**Fig 1:**
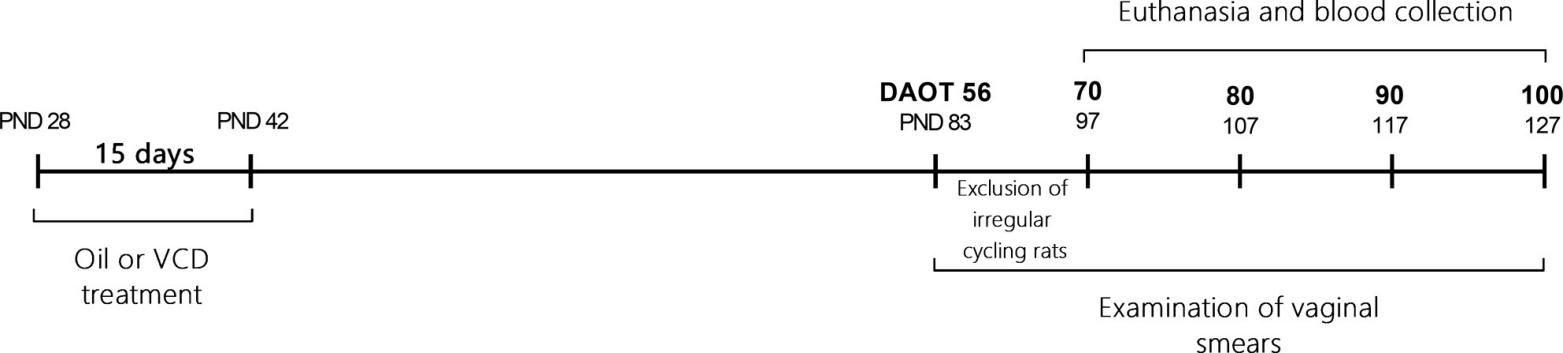
Schematic experimental design showing the timeline of the experimental protocol. Twenty-eight-day-old female rats were treated daily with s.c. injections of VCD or Oil for 15 days. The estrous cycle was assessed daily from day 56 after the onset of treatment until euthanasia. From 56 to 70 days after onset of treatment rats with irregular cycles were eliminated from the study. Rats were decapitated in diestrus at 70, 80, 90 and 100 ± 3 days after the onset of the treatment at 9–11 a.m. Trunk blood was collected for hormones measurements. PND, postnatal day; DAOT, days after the onset of the treatment.

Twenty-eight-day-old female rats received daily s.c. injections of VCD for 15 days. The estrous cycle was assessed daily from day 56 after the onset of treatment until euthanasia, and the irregular cycling rats were removed from the study until day 70 (Fig 1).

Because the transition period between low and higher levels of FSH in this model seems to occur between 60 to 120 days after the onset of VCD treatment [22], the rats were decapitated between 9 and 11 a.m at 70, 80, 90 and 100 ± 3 days after the beginning of the treatment. The experiments were performed on 8 groups of rats (Oil and VCD for each time point), and a total of 90 rats were used.

Trunk blood was collected into heparinized tubes, and plasma was separated for the measurement of the following hormones by radioimmunoassay (RIA) or ELISA: anti-Müllerian hormone (AMH), estradiol (E2), progesterone (P4), follicle-stimulating hormone (FSH), luteinizing hormone (LH), testosterone, dihydrotestosterone (DHT), corticosterone, prolactin, triiodothyronine (T3) and thyroxine (T4).

### Estrous cycle

Vaginal smears were collected every morning between 7 and 9 a.m. from day 56 after the onset of treatment with VCD. The phases of the estrous cycle were determined as described previously [25]. A regular 4-day cycle was defined as a sequence of metestrus, diestrus, proestrus and estrus. The estrous cycle was considered irregular when it did not follow any of the sequence patterns for a 4-day cycle. Long periods of diestrus, metestrus or estrus were the most common alterations (see S1 Table). In our colony, around 20–25% of adult female rats display irregular cycles, which suggest malfunction of the hypothalamus-pituitary-ovarian axis. In the experimental protocol used in this study, it is not possible to determine whether estrous cycles are regular before treatment, as it begins in the period of vaginal opening (28 days of age). In a previous study from our group, we reported that [24, 32], the same percentage of irregular cycles was observed in VCD and oil-treated rats until nearly 85 days after the onset of treatment, showing that the irregularity of the estrous cycle until 85 after VCD treatment onset was not due to an effect of VCD, but rather to a random effect of our colony of rats. Thus, in the present study, the exclusion of irregularly cycling rats was done in both groups, VCD and Oil-treated, in a time window from 56 to 70 days after the treatment onset, after which, regular and irregular cycles were considered as experimental findings.

In addition, although 4-day cycle is the most frequent cycle length in rodents, the 5-day cycle is considered a normal variant of regular cycles. 5-day cycles are lengthened by repeating the diestrus (most common) or estrus phase. Because there is no consensus in the literature on the profile of luteal progesterone secretion in the diestrus phase of 5-day cycles [33–36], we only used rats that exhibited 4-day cycles to avoid conflicting results. It is worth noting that when a 5-day cycle eventually occurs in rats amongst several regular 4-day cycles, the pattern of the cycle was classified as regular.

### Hormonal measurements

Blood samples were centrifuged at 1200 g for 20 min at 4°C, and the plasma was separated and frozen at -70°C until it was assayed. All samples were measured in the same assay to avoid interassay variation. Plasma FSH, LH and prolactin were assayed using a double-antibody RIA method with specific kits provided by the National Hormone and Peptide Program (Harbor-University of California at Los Angeles). The FSH primary antibody was anti-rat FSH-S11, and the standard was FSH-RP2. The antiserum for LH was LH-S10 with RP3 as a reference. The prolactin antiserum and reference preparation were anti-rat PRL-S9 and PRL-RP3, respectively. The lower limits of detection for FSH, LH and prolactin were 0.2, 0.04 and 0.19 ng/mL, and the intra-assay coefficients of variation were 3%, 3.4% and 4%, respectively. Plasma E2, P4 and testosterone were determined using specific kits for humans provided by MP Biomedicals (Costa Mesa, CA, USA). The intra-assay coefficients of variation and the lower limits of detection were 7.2% and 8.6 pg/mL for E2, 7.6% and 0.02 ng/mL for P4 and 9.1% and 0.07 ng/mL for T, respectively. Plasma DHT was determined using the DIAsource Immunoassays kit for humans (Nivelles, Belgium). The intra-assay variation and lower limit of detection for DHT were 5.2% and 20 pg/mL, respectively. Total triiodothyronine (T3) and thyroxine (T4) were determined using Coat-a-Count Siemens kits for humans (Munich, Germany). The intra-assay errors were 4.7% and 3.1%, and the lower limits of detection were 0.06 pg/mL and 2.5 ng/mL, respectively, for T3 and T4. Plasma corticosterone was assayed as described earlier [37]. The primary antibody and standard were provided by Sigma Inc. (USA), and the 3H-labeled hormone was provided by PerkinElmer (Boston, MA, USA). The lower limit of detection was 0.039 ng/mL, and the intra-assay coefficient of variation was 9%. Plasma AMH was measured by an enzyme-linked immunosorbent assay using a specific kit for mice and rat, provided by Ansh Labs (Webster, TX, USA). The lower limit of detection was 0.23 ng/mL.

### Statistical analyses

The statistical significance of the differences was determined by two-way ANOVA followed by a Bonferroni post hoc test using GraphPad Prism 7 software (GraphPad Software, La Jolla, CA). The data are presented as the mean ± SEM. Significance was accepted at P<0.05.

## Results

From 70 to 90 days after the onset of treatment, control as well as VCD-treated rats showed regular 4-day cycles. A small proportion of them showed 5-day cycles amongst other several cycles of 4 days, which was considered normal. In the 100-day group, the percentage of rats showing irregular cycles from 90 to 100 days after the onset of treatment increased. In general, rats extended the cycle, repeating the diestrus or estrus phase. Altogether, the percentage of VCD-treated rats of the 100-day group that presented elongated estrous cycles was 73.3% (11 in 15 rats) versus 33.3% (3 in 9 rats) of the control rats (see S2 Table). Because three rats from the 100-day group were in constant estrus, euthanasia was done at this stage of the estrous cycle in these rats.

**Fig 2:**
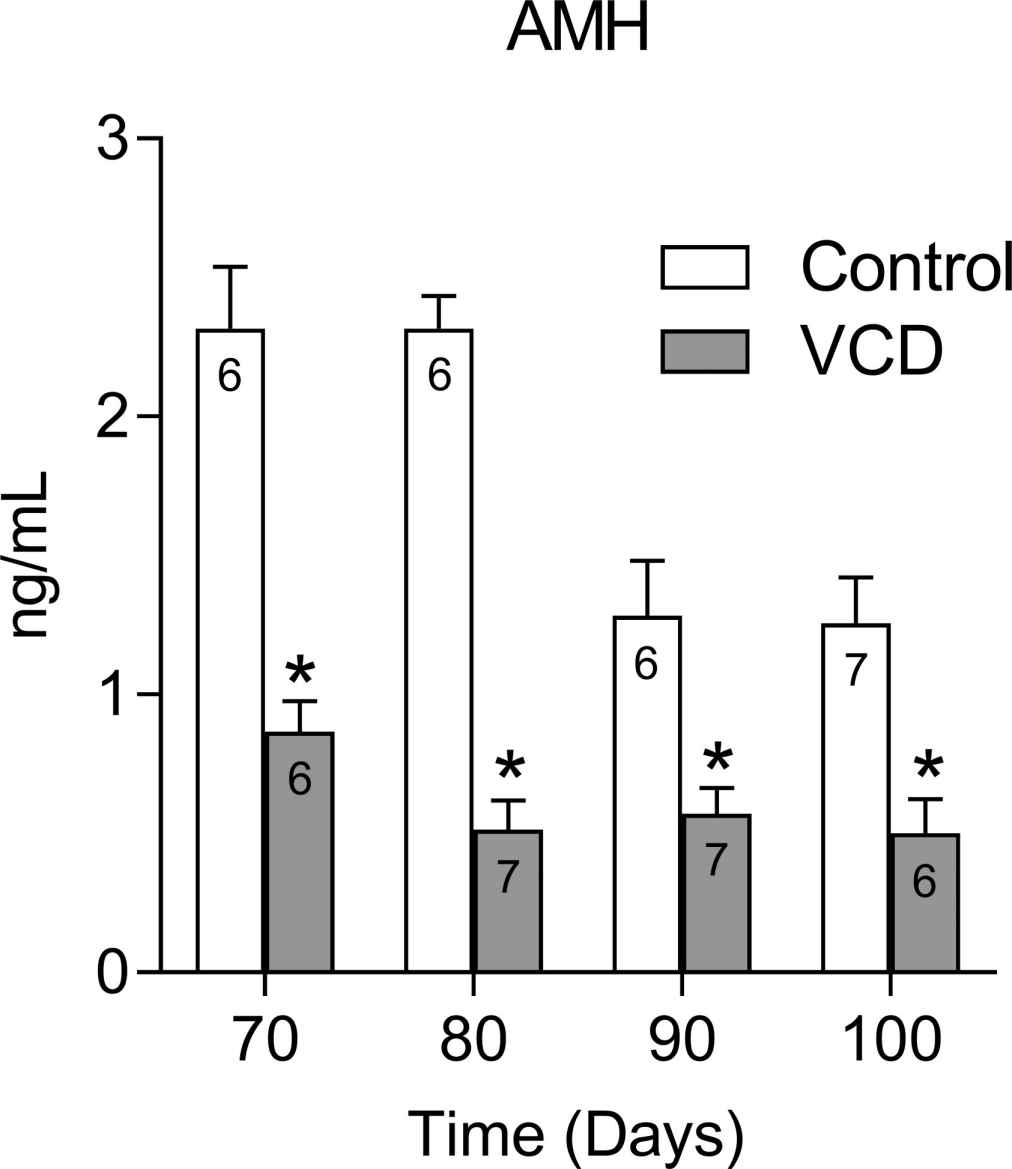
Effect of follicular depletion induced by VCD on plasma AMH. Twenty-eight-day-old female rats were injected daily with VCD or oil s.c. for 15 days. The estrous cycle was monitored from day 56 after the onset of the treatment. The rats were euthanized, and blood samples were collected during the diestrus phase approximately 70, 80, 90 and 100 days after the onset of VCD/Oil administration. *P<0.01 compared to the control rats at the same time points. The numbers inside bars indicate the total number of animals in the respective group.

Fig 2 shows the plasma levels of an ovarian reserve marker, the AMH, of control and VCD-treated rats during all time points studied. There was a significant interaction effect between VCD treatment and time (F (3, 43) = 6.788; P = 0.0008). Plasma AMH was lower at all time points in the VCD-treated rats (P<0.001).

**Fig 3:**
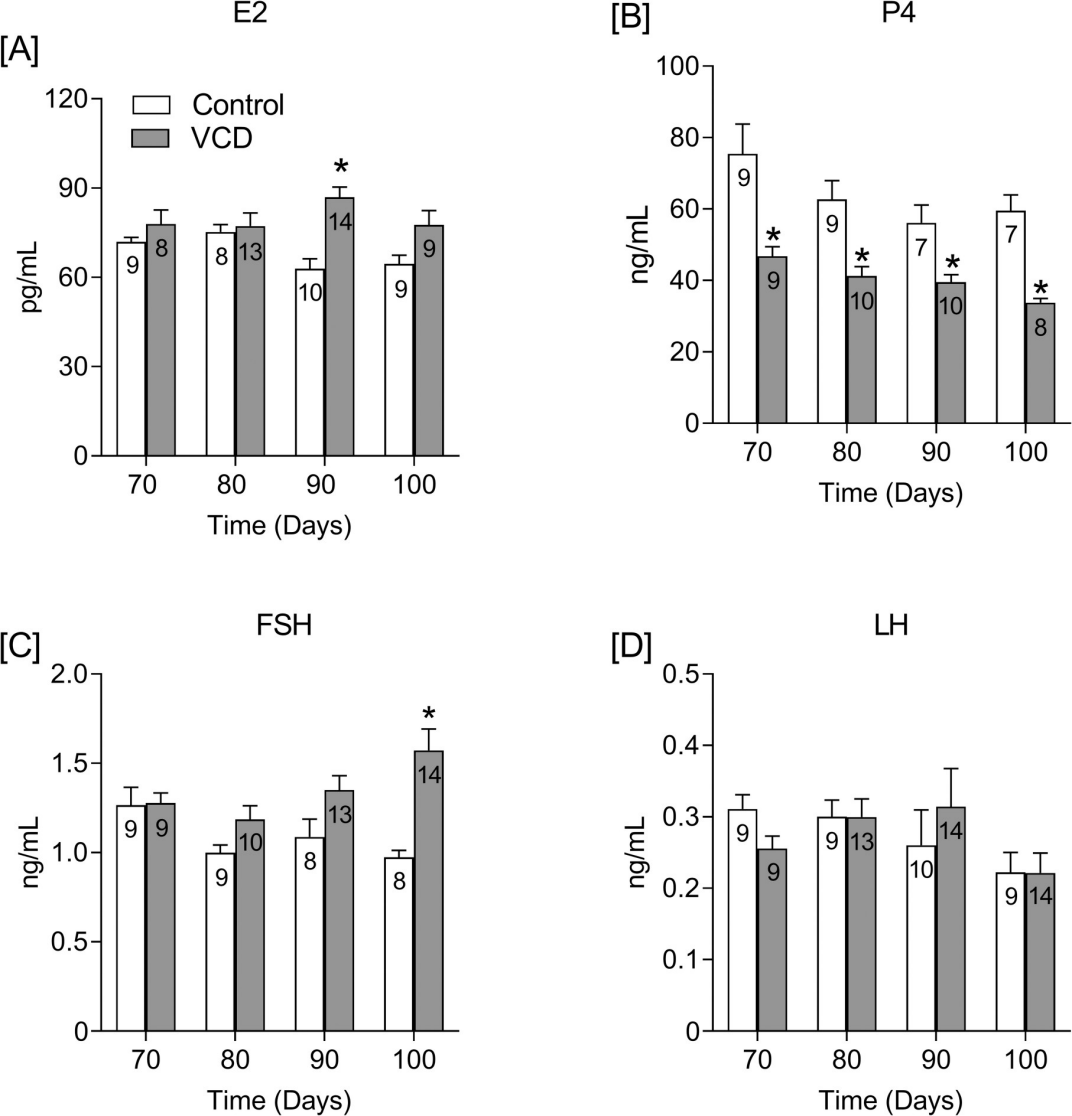
Effect of follicular depletion induced by VCD on plasma E2, P4, FSH and LH. Plasma levels of estradiol (E2; **A**), progesterone (P4; **B**), follicle-stimulating hormone (FSH; **C**) and luteinizing hormone (LH; **D**) from 70 to 100 days after the onset of VCD or Oil treatment. Experimental design is described in Fig 2 legend. *P<0.05 compared to the control rats at the same time points. The numbers inside bars indicate the total number of animals in the respective group.

Regarding estradiol (E2) level, there was a significant effect of VCD (F (1, 72) = 17.46; P<0.0001), and an interaction with time was detected (F (3, 72) = 3.557; P = 0.0184). Compared to the respective control groups, estradiol was higher than control only at 90 days after the onset of VCD treatment (P<0.001; Fig 3A). The level of progesterone (P4) was also affected by VCD treatment (F (1, 61) = 53.05; P = 0.0081) and time (F (3, 61) = 4.297; P<0.0001). Unlike estradiol, plasma progesterone was lower compared to control animals in all of the VCD-treated rats (P<0.05; Fig 3B). Regarding plasma FSH level, there was an interaction between VCD treatment and time (F (3, 72) = 3.596; P = 0.0176). In VCD-treated rats, plasma FSH was higher than in controls only at 100 days after the onset of VCD treatment (P<0.001; Fig 3C), and no effect was detected on plasma LH, which remained unchanged (Fig 3D).

**Fig 4:**
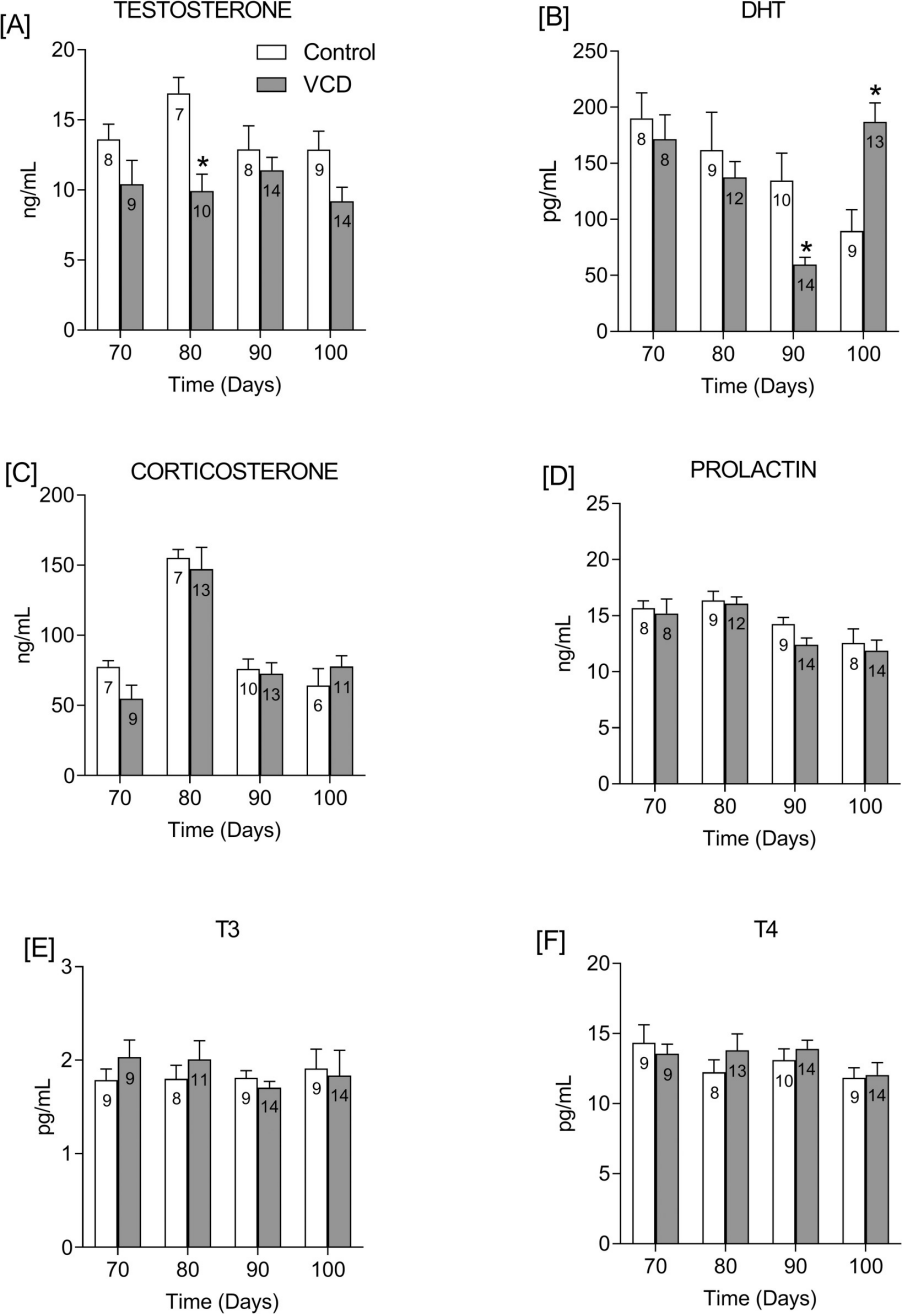
Effect of follicular depletion induced by VCD on plasma androgens, corticosterone, prolactin and thyroid hormones. Plasma levels of testosterone (**A**), dihydrotestosterone (DHT; **B**), corticosterone (**C**), prolactin (**D**), triiodothyronine (T3) and thyroxine (T4) from 70 to 100 days after the onset of VCD treatment. Experimental design is described in Fig 2 legend. *P<0.05 compared to the control rats at the same time points. The numbers inside bars indicate the total number of animals in the respective group.

Plasma testosterone was affected by VCD treatment (F (1, 71) = 18.11; P<0.0001), but there was no effect of time in this secretion. Although a trend towards a decrease in plasma testosterone was seen in all of the times studied in the periestropausal group, statistical significance was detected only 80 days after the onset of the treatment compared to the control group (P<0.01; Fig 4A). Plasma DHT was strongly affected by time (F (3,75) = 5.997; P = 0.001) and by the interaction of VCD treatment and time (F (3, 75) = 7.344; P = 0.0002). DHT progressively decreased in control rats from 70 to 100 days. In VCD-treated rats, DHT also exhibited a progressive drop at 70–90 days, which was significantly lower (P<0.05; Fig 4B) compared to the control group at 90 days. However, at 100 days, there was a significantly sharp increase in DHT in the VCD-treated rats (P<0.01; Fig 4B). There were no differences between control and treated rats in plasma corticosterone, prolactin, T3 or T4 (Fig 4C–4F, respectively).

## Discussion

This study is focused on the reproductive cycle and endocrine changes during a narrow time window of the transitional period to the ovarian failure in an ovarian-intact perimenopausal rat model using VCD. Herein, we showed that VCD-induced follicular depletion resulted, in the diestrus phase, in a lower AMH and progesterone plasma levels along with normal estradiol, LH and FSH plasma levels and no changes in estrous cyclicity, from 70 to 90 days after the onset of VCD treatment, which may correspond to the early/mid-transition period to estropause. On the other hand, the increased FSH and DHT plasma levels along with irregular/elongated cycles in the 100-day VCD-treated rats seem to signal the end of this transition. Below, we discuss the differences and similarities between this animal model and women.

### Reproductive cycle

In women, the menopausal transition is generally associated with irregular menstrual cycles [30, 38], nonetheless, regular ovulatory cycles may occur throughout the transition period [39, 40]. The most striking difference between rats and women is that unlike women, female rats continue to cycle for a longer time. In the rat, the regular cycles gradually change to irregular/elongated cycles in middle-aged rats. Irregular cycles initiate on average at 8 months of age, followed by a period of constant estrus (CE; high estrogens) initiated at 10 mo. of age, and then by a period of persistent diestrus (PD; low estrogens and progesterone), at 17 mo. of age. Nearly 60% of rats skip CE, remaining in irregular cycles until they enter PD; however, half of them continue to cycle irregularly until 22 mo. of age [5, 6, 41].

In this study, the majority (over 70%) of the VCD-treated rats showed an increased cycle length by increasing diestrus or estrus phase in the last one or two cycles in the 100-day group (see S2 Table). Since it was stated by Chakraborty and Gore (5) that estropause is characterized initially by irregular, usually prolonged, estrous cycle, we might consider that the rats in the present study were evaluated from the period preceding (70–90 days) to the onset (90–100 days) of VCD-induced estropause. Thus, the term “periestropause” might be considered for this transition period in this animal model.

Interestingly, the elongation of the estrous cycles coincided with the first significant increase in FSH plasma levels, which in turn, was found in association with decreased progesterone and AMH and normal estradiol plasma levels, suggesting that the onset of VCD-induced estropause is in some extent similar to the typical hormonal changes found in the late-perimenopausal period in women. Thus, VCD treatment seems to anticipate the transition period of natural aging from middle-age (8–12 mo.) to young adult (approx. 4 mo.) in a very narrow period of time ranging from 90 to100 days after the onset of treatment, rending a temporal resolution that facilitates experimental design.

### Anti-Müllerian Hormone (AMH)

It is well known that AMH levels correlate directly with the decrease in ovarian follicle numbers and have been described as the most reliable marker of ovarian follicle reserve [42–44]. In women, AMH levels decrease in an age-dependent manner from 18 to 50 years and AMH remains very low or undetectable after menopause [42]. In VCD-treated monkeys (*Macaca fascicularis)* and mice, a linear correlation has been demonstrated between low levels of AMH and decreased number of growing follicle [43], showing a causal effect of VCD in decreasing AMH plasma levels by reducing ovarian follicle reserve. Thus, in the present study, we used AMH plasma levels as an index of ovarian follicle reserve. Herein, we showed that AMH plasma levels in the 70/80-day control groups presented AMH levels around 2.3ng/mL, compatible with those already found in rats [44], while VCD-treated groups showed an expressive reduction in all times studied. Although we have not evaluated the population of ovarian follicles, the low and practically constant levels of AMH suggest that follicular depletion has not changed dramatically over the short period studied. The very low levels (around 0.45 ng/mL) of AMH in the 100-day group appear to be in agreement with the 80% decrease in primary and preantral follicles number 120 days after the onset of VCD treatment as previously described by Mayer and cols [22].

### Estradiol

Although the decline in the number of ovarian follicles has been related to estrogen hyposecretion, it has been shown recently that the association of perimenopause with estrogens deficiency is erroneous. In fact, perimenopause is characterized by normal or even elevated levels of estrogens [28, 45–47]. Moreover, luteal estrogen levels may vary from normal to high from one cycle to another in perimenopausal women [47]. Since in the present study all rats were studied in diestrus (late luteal phase), the normal or high estradiol plasma levels found in our study is in good agreement to the observed in women. In VCD-treated animals, previous studies have shown estradiol plasma levels is increased, decreased or unaltered in mice depending on the experimental protocol [14, 15, 18, 19]. In monkeys, estrogen plasma levels are slightly higher four months after the onset of VCD treatment [48] and in rats, it remains unaltered for long periods (1–20 months) after treatment [26, 27].

Based on our previous observation that VCD-treated rats have fewer antral follicles [24], our data suggest that, despite follicular depletion, the ovaries seem to be able to produce estradiol during the transition period, in part, by the remaining follicles lacking oocytes that still retain functional granulosa cells [49]. Additionally, extra ovarian androgen conversion to estradiol, such as in adipose tissue, might be contributing for the maintenance of estradiol plasma levels in this period. Taken together, our results reinforce current clinical findings and weaken the paradigm of estrogen deprivation.

### Progesterone

In contrast to estradiol, progesterone declines during the transition period. Although lower progesterone has been consistently reported by several authors [45, 50] and is one of the most striking and reproducible features of perimenopause, medical research has remained focused almost entirely on estrogens. In VCD-treated mice, progesterone levels have been evaluated in estropause [14, 23] or in proestrus [15], making difficult the comparison with our results, since our study was carried out in diestrus. In the present study, progesterone was lower in VCD than in control rats at all of the studied times along with normal gonadotropins levels, indicating that progesterone might be an earlier sign of ovarian failure. Reduction in progesterone secretion has been associated with mood changes observed during late luteal phase [51]. Interestingly, mood changes, such as depression and increased anxiety, emerge years before menopause in temporal association with the decrease in progesterone secretion that occurs several years prior the last menstrual period [45]. This association in mood changes with low progesterone was recently demonstrated in a study by our group, which demonstrated that VCD-induced periestropausal rats exhibited decreased progesterone and increased anxiety in diestrus [24]. In addition, the increased ratio of estradiol/progesterone may also contribute to physical symptoms, such as heavy flow, which is one of the most stressful and frequent symptoms reported by women in perimenopause [52].

Although lower progesterone is the clearest endocrine feature of the transition period to menopause, the mechanisms underlying this change are still unclear. Three explanations why perimenopausal women exhibit lower levels of progesterone have been proposed: 1) lower secretion of progesterone in a normal/ovulatory cycle; 2) a shortened luteal phase length within the ovulatory cycle; and 3) more frequent anovulatory cycles [53]. In the present study, the low progesterone levels in regularly cycling rats (70-90-day groups) reinforce the first hypothesis, and suggest an impaired secretory activity of the corpora lutea. In contrast, it is possible that rats with irregular cycles (100-day VCD group) are not ovulating properly and therefore not producing enough corpora lutea, which is consistent with the third hypothesis. Therefore, the similarities between the progesterone data of VCD-induced periestropausal rats and the data found in women support the importance of this animal model. Moreover, the reductions in plasma progesterone, rather than hypoestrogenism, could explain the symptoms of perimenopausal women.

### FSH and LH

In women, a hallmark of menopause is the increase in plasma FSH [28]. However, during perimenopause, while some studies show higher levels of FSH [30, 45, 54, 55] others show that during most of the perimenopausal period, FSH remains unchanged, whereas it increases only during late perimenopause [28, 29], which may be associated with prolonged menstrual cycles at the end of perimenopause [56]. Accordingly, our results show that between 90 and 100 days after the onset of VCD treatment, the last two cycles became irregular and longer as FSH increased.

Several studies evaluated FSH in mice and found higher FSH 35 days after the onset of VCD treatment [14–18]. It is worth noting that the effects of VCD on mice occur earlier than in rats [14, 21, 22], and therefore, they should not be compared temporally. In rats, FSH is higher from 4 months after the onset of VCD treatment [22, 26, 27]. However, in these studies, FSH was evaluated once a month or at even longer intervals, which made it difficult to pinpoint the FSH turning point in this transition period of the VCD model. In the present study, however, it was possible to demonstrate that this turning point occurred sometime between 90 and 100 days after the onset of VCD treatment, along with the elongation of the estrous cycle, defined as estropause. Therefore, this period seems to correspond to the late perimenopause in women for which the term “late periestropause” might be considered for this animal model.

The increase in FSH secretion could be explained by the decrease in inhibin B secretion, which results in increases in FSH secretion. In fact, a similar study reported a significant and inverse correlation between inhibin B and FSH [27]. Since we observed an increase in FSH secretion at 4.2 months of age (100 days after the onset of VCD treatment), it is reasonable to assume that the turning point in inhibin B secretion also occurred in this period. Similarly, a rise in FSH secretion is also associated with a decrease in inhibin B in women [57]. Thus, the hypothesis that VCD-induced follicular depletion may have decreased inhibin B secretion and consequently specifically increased FSH secretion is plausible.

Although in women some authors found high levels of LH during perimenopause [30], there is a strong consensus that in most of the perimenopause period, LH remains at a basal level while FSH increases [38, 39, 45, 55, 56]. Accordingly, in our study, increased FSH was observed at 100 days, while no change in LH secretion was seen. The lack of change in LH plasma levels in our model is expected based on normal estradiol secretion and corroborate the abovementioned clinical findings.

### Testosterone

Although some studies that followed women throughout their lives found an increase [58] or no difference in testosterone levels [59], other studies showed that the testosterone level in women in their forties is half of the level in their twenties [60, 61]. However, the level of testosterone does not seem to be affected by age, since the decrease in testosterone in the years preceding menopause is followed by an increase right after menopause [62, 63]. In our study, although testosterone was significantly lower only at 80 days after the onset of VCD treatment, it tended to be lower in all of the other studied periods, including at 100 days, which was considered here as the late period of the transition period to estropause induced by VCD. Interestingly, a positive correlation has been found between AMH and androgen levels in women of reproductive age, which suggests a synchrony between granulosa and theca cells, producing AMH and androgens, respectively [42]. On the other hand, in women, this correlation seems to be lost after menopause when higher amounts of testosterone are secreted by the stromal cells [64] while AMH becomes undetectable [65]. Thus, the low levels of both, AMH and testosterone, in VCD-treated rats in the present study, strengthen the hypothesis that the period study precedes ovarian failure.

### Dihydrotestosterone (DHT)

Testosterone, secreted from the ovaries and the adrenal glands, can be peripherally metabolized by 5-alpha-reductase to DHT. We observed that this androgen tends to be lower compared to control rats from 70 to 90 days after the onset of VCD treatment. However, in the 100-day group, DHT was significantly higher than the control group while testosterone remained lower, which suggested a higher activity of 5-alpha-reductase. Indeed, the ovaries in postmenopausal women are hormonally active and continue to produce significant amounts of androgens for many years after menopause, and postmenopausal ovaries secrete more testosterone than premenopausal ovaries, which suggests that the elevated gonadotropin levels would be responsible for the stimulation of stromal androgen-secreting cells [62]. Our data show that although testosterone tended to be lower in VCD treated rats at all of the times studied, DHT increased at the same time as FSH, at 100 days after the onset of VCD treatment. Although we cannot state that there is a causal relationship between an FSH and a DHT increase, it is known that FSH can increase 5-alpha-reductase activity [66, 67], which increases DHT synthesis from testosterone. Since increase in DHT is correlated with hair growth, higher levels of this hormone could be involved in the hirsutism observed in older women. Another important observation is that an increased DHT/estradiol ratio favors follicle atresia and promotes anovulation, which partially occurred due to the ability of DHT to block the FSH-induced LH receptor expression in granulosa cells [68]. These data fit well with the irregular cycles seen in the 100-day group.

### Corticosterone

In women, cortisol increases with age, but no differences are observed between the stages of the menopause transition [69]. However, in women with severe vasomotor symptoms, higher urinary cortisol has been observed at the end of perimenopause and early menopause [69]. It has been suggested that the increased cortisol levels in overnight urine can be due, at least in part, to the increase in cortisol secretion after a hot flash [70].

In diestrus rats, corticosterone secretion increases with age [71]. Since both groups (control and VCD-treated) are around the same age, the lack of differences in corticosterone levels between controls and VCD-treated rats suggests that ovarian failure is not directly involved in hipothalamic-pituitary-axis (HPA) control during this transitional period. In fact, the basal level of corticosterone 80 days after the onset of treatment is not different from control rats [24]. Regarding the increased levels of corticosterone in the 80-day group, we have no explanation, since the time interval from 9:00 to 11:00 a.m. was never exceeded and rats from different groups (70-100-day) were decapitated each day, the final number of animals in each group being gradually reached at the end of the experiments.

### Prolactin

We did not observe any changes in prolactin secretion at any of the times that were studied. In women, prolactin does not change during the perimenopause period [45, 56, 63]. Prolactin secretion is estrogen-dependent, and in middle-aged rats, the estradiol-induced prolactin surge is not different from that in young rats [72]. Thus, as estrogen levels remain unchanged during the transition period in women as well in rats, it is reasonable to expect that prolactin would also remain stable in both species. In women, this effect of estradiol was confirmed by the low levels of prolactin during postmenopause, which correlated with persistent hypoestrogenism [73].

### T3 and T4

There is a mutual relationship between the thyroid and ovaries, i.e., thyroid hormones affect the female reproductive system, and ovarian hormones affect thyroid function during fertile periods, as well in menopause [74, 75]. Although thyroid diseases are more common in women [76], and hypothyroidism increases with age [77], thyroid hormones during perimenopause are poorly investigated. During the menopausal transition, it was found that 9.6% of women had altered TSH values, but no association was found with reproductive hormone concentrations or with any other menopausal symptoms other than bleeding length and a feeling of fearfulness [78]. On the other hand, other studies found no difference in TSH levels across menopausal transition [63, 79], suggesting normal thyroid function. Accordingly, our data show that plasma T3 and T4 remained unchanged throughout the studied period in both controls and VCD-treated rats, which suggests that hypothalamus-pituitary-thyroid axis seems to be unaffected in this model of perimenopause.

## Conclusions

This is the first study attempting to establish a period of perimenopause in the VCD rat model that corresponds to that in women based on the estrous cycle and a broad endocrine profile. We have shown here that, by shifting the primary cause of reproductive senescence from brain to ovary, VCD anticipated irregular/elongated cycles, characteristic of estropause in rats, which occurred concurrently with an increase in plasma FSH. Elongated cycles associated with increased FSH and DHT seems to signal the final stage of the transition to estropause, resembling the late-perimenopause period in women. On the other hand, decreased plasma AMH associated with decreased progesterone and normal estradiol, FSH and LH resembles the early/mid-perimenopausal period in womenBased on these data, we may conclude that the period from 70 to 100 days after the onset of VCD treatment is an adequate window of time for studies of the transitional period of reproductive function in female rats, for which the term “periestropause” might be considered. Thus, the present work provides an important temporal and endocrine basis to guide research in this perimenopausal animal model.

## Supporting information

S1 Table Daily analysis of the estrous cycle phases of the animals excluded from the experiment. M, Metestrus; D, Diestrus; P, Proestrus; E, Estrus. Two letters together indicate a transitional smear. Rats with irregular as well as 5-day cycles were eliminated from the study, before the onset of the experiments. A regular 4-day cycle was considered as a sequency of phases M or D, D, P and E [33]. Although the vast majority of rats have regular 4-day cycles, from vaginal opening to 6 months of age, a small portion of rats exhibit 5-day cycles, which were included in this table. Another portion of rats exhibited longer cycles with repeated phases of M, D or E, or were even acyclic and were considered as irregular and the rats were excluded before the onset of the experiments. N = 25.
(XLSX)

S2 Table Daily analysis of the estrous cycle phases of the animals of 70, 80, 90 and 100-day groups. M, Metestrus; D, Diestrus; P, Proestrus; E, Estrus. Shaded cells indicate the day of euthanasia. Two letters together indicate a transitional smear. Rats with irregular as well as 5-day cycles were eliminated from the study. When a 5-day cycle eventually occurs in rats amongst several regular 4-day cycles, the whole cycle was classified as regular [33]. Vaginal smears initiated 56 days after the onset of VCD/oil treatment. In order to avoid too long tables (from 56 to 102 days after the onset of treatment), we only presented the cycle from the beginning in the 70-day group, although in all animals the cycle was analyzed since day 56. **Irregular cycles at 100-day groups.
(XLSX)

S1 File Checklist. (PDF)

S1 Fig Fig 2 data presented as scatter plot. (TIF)

S2 Fig Fig 3 data presented as scatter plot. (TIF)

S3 Fig Fig 4 data presented as scatter plot. (TIF)
