# Supplementary figures and images for: Endocrine profile of the VCD-induced perimenopausal model rat
Source: PLoS One. 2019 Dec 30;14(12):e0226874. doi: 10.1371/journal.pone.0226874 (PMC6936812; doi:10.1371/journal.pone.0226874)

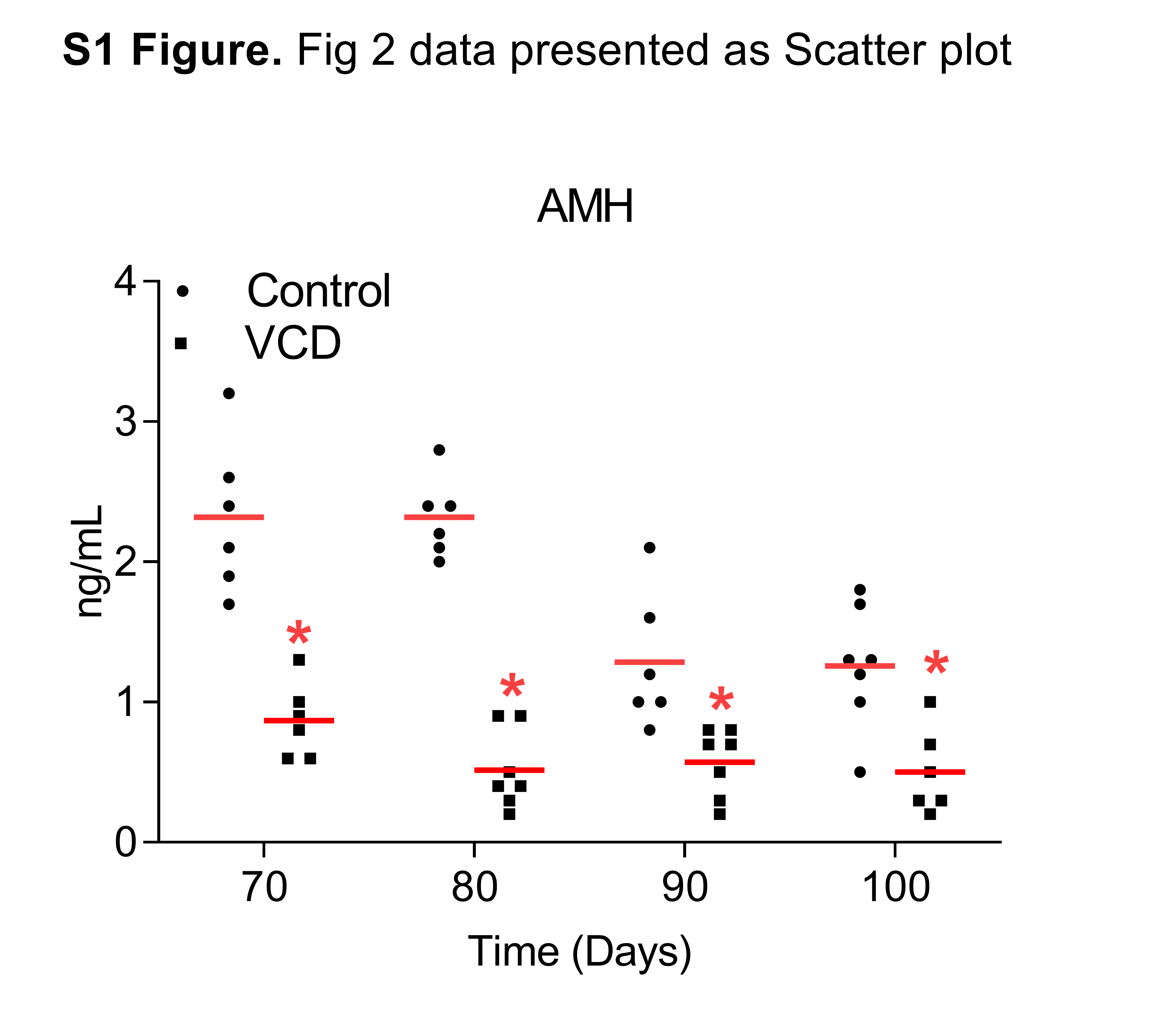

Supplement: S1 Fig — (TIF) [file pone.0226874.s004.tif]

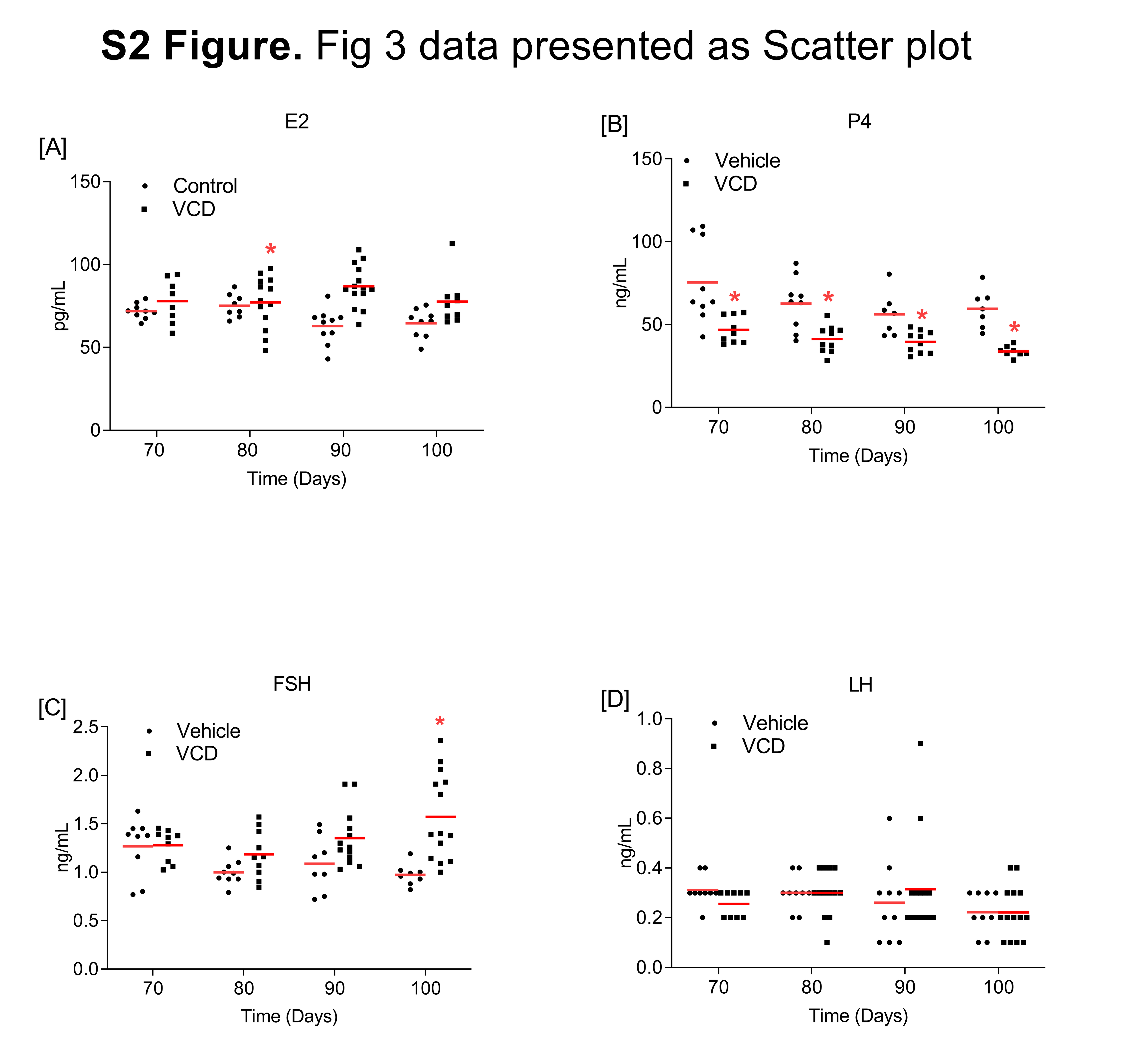

Supplement: S2 Fig — (TIF) [file pone.0226874.s005.tif]

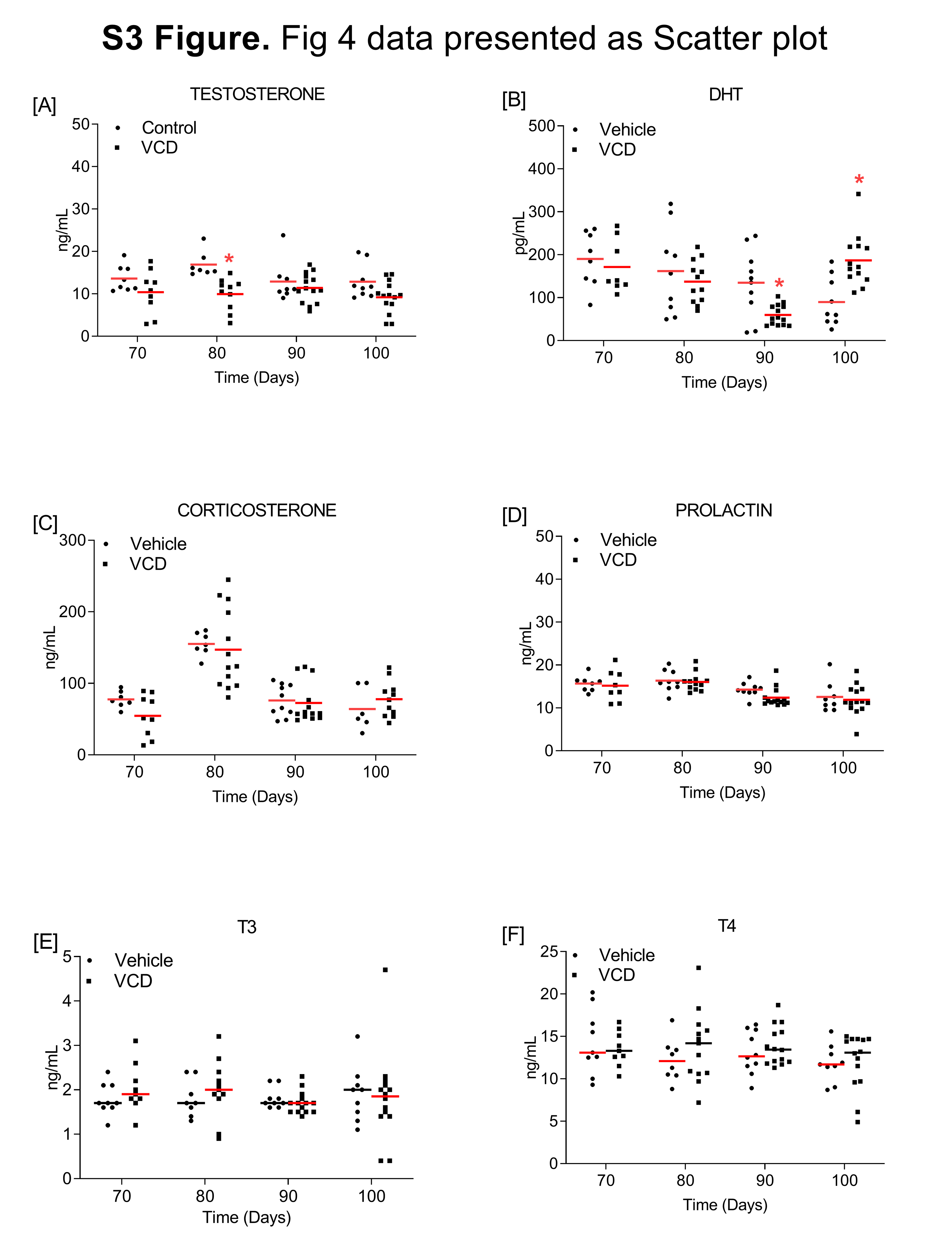

Supplement: S3 Fig — (TIF) [file pone.0226874.s006.tif]
